# Pharmacological effects of Safranal: An updated review

**DOI:** 10.22038/IJBMS.2023.69824.15197

**Published:** 2023

**Authors:** Danial Esmaealzadeh, AmirAli Moodi Ghalibaf, Mohammad Shariati Rad, Ramin Rezaee, Bibi Marjan Razavi, Hossein Hosseinzadeh

**Affiliations:** 1 School of Medicine, Mashhad University of Medical Sciences, Mashhad, Iran; 2 Student Research Committee, Faculty of Medicine, Birjand University of Medical Sciences, Birjand, Iran; 3 International UNESCO Center for Health-Related Basic Sciences and Human Nutrition, Faculty of Medicine, Mashhad University of Medical Sciences, Mashhad, Iran; 4 Applied Biomedical Research Center, Mashhad University of Medical Sciences, Mashhad, Iran; 5 Targeted Drug Delivery Research Center, Pharmaceutical Technology Institute, Mashhad University of Medical Sciences, Mashhad, Iran; 6 Department of Pharmacodynamics and Toxicology, School of Pharmacy, Mashhad University of Medical Sciences, Mashhad, Iran; 7 Pharmaceutical Research Center, Pharmaceutical Technology Institute, Mashhad University of Medical Sciences, Mashhad, Iran

**Keywords:** Crocus sativus, Patent, Review, Saffron, Safranal

## Abstract

Safranal (a monoterpene aldehyde) is the major volatile component of saffron which is responsible for the saffron unique odor. Several studies have shown the pharmacological activities of safranal including anti-oxidant, anti-inflammatory, cardioprotective, neuroprotective, nephroprotective, gastrointestinal protective, etc. This study was designed to review the pharmacological and medical effects of safranal and up-to-date previous knowledge. Moreover, some patents related to the pharmacological effects of safranal were gathered. Therefore, electronic databases including Web of Sciences, Scopus, and Pubmed for pharmacological effects and US patent, Patentscope, and Google Patent for patents were comprehensively searched by related English keywords from 2010 to June 2022. According to our review, most of the studies are related to the safranal effects on CNS such as antianxiety, analgesic, anticonvulsant, antiischemic, anti-tremor, memory enhancement and its protective effects on neurodegenerative disorders such as Alzheimer’s, Parkinson and Huntington diseases. Other effects of safranal are antiasthmatic, antihypertensive, antiaging, anticataract, etc. Moreover, the protective effects of this agent on metabolic syndrome and diabetic nephropathy have been shown. Different mechanisms including anti-oxidant, anti-inflammatory, muscle relaxation, antiapoptotic, and regulatory effects on the genes and proteins expression related to signaling pathways of oxidative stress, inflammation, apoptosis, proliferation, etc. are involved in safranal pharmacological effects. Some patents for the prevention and/or treatment of different diseases such as liver cancer, sleep disorder, depression, cognitive disorder, obesity and PMS were also included. Based on the documents, safranal is considered a promising therapeutic agent although more clinical studies are needed to verify the beneficial effects of safranal in humans.

## Introduction

Saffron (red gold or* Crocus sativus* L. from the family Iridaceae), is one of the most expensive natural products that is broadly cultivated in countries such as Azerbaijan, China, Egypt, France, and Turkey, and especially Iran for more than 4000 years ago. The plant has been mentioned in the medical book “Al-Havi” written by Zakariya Razi (1-3). The dried stigma of this perennial herb is commonly used as a flavoring and coloring food additive and, less extensively, as a perfume ingredient (4). Moreover, saffron is an enriched pool of bioactive compounds including crocetin, crocins, picrocrocins, safranal, essential oils, minerals, and trace amounts of B family vitamins which have attracted the attention of herbal medicines (5, 6). Saffron has been traditionally used because of its therapeutical effects for the treatment of many diseases in different traditional health systems. These therapeutic effects are attributed to the occurrence of crocetin, safranal and crocin as saffron main constituents (7, 8).

Safranal (2,6,6-trimethyl 1,3-cyclohexadien 1-carboxaldehyde) constitutes 30-70% of volatile compounds of saffron and 0.001-0.006% of saffron dry matter. It is responsible for the odor of saffron (9-12). The amount of safranal in Iranian saffron is 0.06–0.29 mg/g (13). The compound is produced from picrocrocin. The slow degradation of picrocrocin in aqueous extracts of saffron at different temperatures (5–70 ^0^C) has been observed via second-order kinetics. Purified picrocrocin in 100 ^0^C of water shows high stability and safranal generation in the first 5 h was confirmed (14). Besides, safranal has been reported to occur in other herbal plants such as *Centaurea sibthorpii *(15),* Centaurea amanicola *(16)*, Centaurea consanguinea *(16)*, Erodium cicutarium *(17)*, Citrus limon *(18), etc. 

In our previous article, we reviewed the pharmacological effects of the safranal such as anti-inflammatory, anti-oxidant, anticonvulsant, antidepressant, and other medical activities (19). The present study reviews the pharmacological and medical effects reported for safranal in the period 2010-2022 and presents some patents registered based on their therapeutic values.

***Method of search***

Electronic databases including Web of Sciences, Scopus, and Pubmed were comprehensively searched for pharmacological effects of safranal and the US patent, Patentscope, and Google Patent for safranal patents for items published from 2010 to June 2022, using “safranal” in the title, abstract and keywords. Also, the references of the retrieved articles were manually checked.

***Anti-oxidative and anti-inflammatory effects***

Several studies have shown the anti-oxidant and anti-inflammatory effects of safranal. It was reported that intraperitoneal injection of safranal (0.5 mg/kg/day) for one month in old rats suppressed age-induced oxidative damage and improved the anti-oxidant defense system. Safranal more likely acts as an indirect anti-oxidant by being hormetic via causing mild oxidative damage which induces anti-oxidative enzymes (20). Various natural compounds such as polyphenols and flavonoids are considered hormetins that act through one or more pathways of the stress response (21). Moreover, some anti-oxidants have prooxidant activities that can induce anti-oxidative defensive responses and finally, show beneficial effects (22). 

In another study, the anti-oxidant and anti-inflammatory effects of safranal on carbon tetrachloride (CCl_4_)-induced inflammation and oxidative stress in rats were evaluated; results demonstrated that relatively high doses of safranal (50 and 100 mg/kg, given orally for 14 days), could improve the anti-oxidant system in the liver. Interleukin (IL) 1-β levels were not affected by safranal but high-dose safranal significantly decreased IL-6 and tumor necrosis factor α (TNF-α) cytokines in the liver (23).

Moreover, safranal (100 mg/kg/day, orally) could reduce the oxidative stress damage induced by sub-chronic inhalation of thinner in different tissues of rats. This was probably achieved by lowering malondialdehyde (MDA) levels in all tissues except the kidneys’ nitric oxide (NO) metabolites, and total oxidant capacity (TOS). Furthermore, safranal increased glutathione (GSH) and total anti-oxidant capacity (TAS) (24).

In preclinical experiments, safranal could function as a potential anti-inflammatory agent in many inflammatory diseases and its effects are mediated by inhibiting NLRP3 inflammasome and reducing IL-1β secretion (25). *In vitro*, it was revealed that safranal inhibited the production of NO, inducible nitric oxide synthase (iNOS), and cyclooxygenase (COX-2) in RAW264.7 and primary BMDM (Bone-marrow-derived macrophage) cells. Safranal also decreased IL-6 and TNF-α mRNA expression and suppressed mitogen-activated protein kinase (MAPK) and nuclear factor-kappa B (NF-ĸB) protein expression in RAW264.7 cells. (26).

Based on the above-mentioned studies, safranal exhibits anti-oxidant effects through increasing the anti-oxidant defense system (by acting as a hormetin), lowering MDA level, increasing GSH content, etc. In addition, through COX-2 inhibition as well as decreasing the levels of Il-6, Il-1β and TNF-α, safranal could act as an anti-inflammatory agent. 

***Cardiovascular protective effects***

Extensive evidence exists on safranal antihypertensive, anti-ischemic and relaxant effects that are mediated through anti-oxidants, regulation of Ca^2+^ homeostasis, inhibition of smooth muscle cell (SMC) contraction and antiapoptotic effects in the cardiovascular system. In rats, safranal produced anti-hypertensive effects in desoxycorticosterone acetate (DOCA) salt-induced hypertension; chronic administration of safranal (1, 2 and 4 mg/kg/day) could reduce mean systolic blood pressure in DOCA salt-induced hypertensive rats but not in normotensive animals in which antihypertensive effects of safranal did not persist (27). This article suggested that the antihypertensive effects of safranal may be caused by blocking calcium channels and relaxing smooth muscle cells (28), affecting gamma-aminobutyric acid (GABA)-benzodiazepine receptor complex (29, 30) or diuretic activity of saffron (31). The antihypertensive activity of the highest dose of safranal was comparable to spironolactone used as a positive control in this study (27). 

The relaxing effects of safranal have been shown in another study conducted on isolated rat aorta contracted by phenylephrine and potassium chloride (KCl). Its maximal relaxation was more than 100% and this effect was not suppressed by incubating aortic rings with NG-Nitro arginine methyl ester (L-NAME) or indomethacin, or following endothelium removal, indicating that this activity is mainly independent of the endothelium. L-type calcium channel blockage and inhibition of smooth muscle contraction are the primary mechanisms of safranal-induced relaxation which are partly related to the endothelium (32).

Safranal effects on damaged cardiomyocytes that underwent hypoxia/reoxygenation (H/R) were investigated; in comparison to the H/R group, safranal-treated H9C2 myoblasts showed significantly higher viability, lower reactive oxygen species (ROS) levels and higher activities of matrix metalloproteinase (MMP) and anti-oxidant enzymes such as catalase (CAT), superoxide dismutase (SOD), glutathione peroxidase (GSH-px). Also, the levels of creatine kinase-MB (CK-MB), lactate dehydrogenase (LDH), MDA and intracellular Ca^2+^ concentration were significantly decreased by safranal. Besides, safranal reduced cleaved caspase-3 and Bax protein expression levels but upregulated Bcl-2 protein expression, and PI3K/GSK3β/AKT signaling. The protective effects of safranal were suggested to be mediated by the inhibition of oxidative stress and apoptosis (33). 

Another study evaluated the safranal effects on isoprenaline-induced myocardial infarction (MI) in rats. The model was induced by isoprenaline subcutaneous injection (85 mg/kg/day) on the 8th and 9th days of the investigation. Safranal could reduce CK, LDH and MDA levels, intracellular calcium concentration and ROS content, and increase serum SOD activity. The recovery of morphologic myocardial changes was also observed following safranal treatment. Therefore, safranal through inhibition of oxidative stress, myocardial contracture and regulation of Ca^2+^ homeostasis, protected the animals against isoprenaline-induced MI (34).

***Renal protective effects***

Safranal has shown nephroprotective through its anti-oxidative and anti-inflammatory properties, in models of diabetic nephropathy as well as nephrotoxicity induced by chemotherapy. It was reported that four-week administration of safranal to diabetic rats improved renal dysfunction (diminished serum blood urea nitrogen (BUN) and creatinine (Cr)) and histopathological damage by its anti-oxidant (via increasing TAS and GSH and decreasing NO and TOS) and anti-inflammatory properties (via decreasing TNF-α, IL-1β, and interferon-gamma (IFN-ϒ)) (35).

In another study, safranal effects on cisplatin-induced nephrotoxicity in rats have been shown. Cisplatin causes significant renal failure with augmented Cr and urea levels. Safranal administration (200 mg/kg, orally, for one month) ameliorated biochemical nephrotoxicity indices in both plasma and renal tissue. Pretreatment of safranal showed a more marked response. Therefore, safranal protected cisplatin-induced nephrotoxicity and oxidative stress (36).

***Effects on respiratory tract***

Besides its anti-oxidative and anti-inflammatory effects, safranal has antitussive and smooth muscle relaxant effects mediated by inhibiting the histamine (H_1_) receptors and stimulating β-adrenoceptors (37). Previous *in vivo* and *in vitro* studies indicated these effects of safranal in different doses of 0.2, 0.5, and 0.75 ml/kg (38), 0.63, 1.25, and 2.5 μg/ml (39), and 1.25 and 2.5 µg (40) 

Sadiq and Zalzala (2021) indicated that safranal has a protective effect on lipopolysaccharide (LPS) -induced acute lung injury (ALI) in mice (41). Treatment with 150 mg/kg safranal caused a remarkable decrease in TNF-α, immunoglobulin E (IgE), and IL-33 levels and differential cell count in ALI. Also, 300 mg/kg safranal reduced the levels of differential and total cell count, lung wet: dry weight ratio, and IgE level, resulting in improved ALI. In all safranal-treated groups, histopathological scores demonstrated a remarkable reduction of inflammatory signs and a significant increase in GSH when compared to the LPS group.

Bukhari et al stated that 0.5 ± 0.067 mg/g safranal reduced oxidative stress and prevented epithelial cell damage during allergic airway inflammation in asthmatic mice (42). Similarly, another study on streptozotocin (STZ)-diabetic asthmatic mice indicated that safranal (0.25, 0.50, and 0.75 mg/kg/day) not only could reduce the level of MDA and NO but also could effectively prevent lung distress by amelioration of oxidative damage. Safranal in diabetic mice increased the level of GSH and the activity of CAT, and SOD (43, 44). Furthermore, both low-dose (200 mg/kg) and high-dose (500 mg/kg) safranal reduced serum IgE level, NF-κB, and the number of mast cells, and normalized the T helper 1 (Th1)/ T helper 2(Th2) cytokine ratio in mice with ovalbumin (OVA)-induced asthma (45). Also, Boskabady et al determined the preventive effects of safranal (4, 8, and 16 µg/mL in drinking water) on tracheal responses and serum cytokine, total nitrite and NO levels as well as increased Th1/Th2 balance in guinea pigs (37).

In another study, the effect of safranal at concentrations of 4, 8 and 16 μg/ml on serum levels of endothelin-1 (ET-1) and total protein (inflammatory markers) in OVA-sensitized guinea pigs was investigated. Safranal was significantly effective in reducing these factors, but its anti-inflammatory effect was reduced at higher concentrations. These findings showed that safranal exhibited a more potent effect than dexamethasone and could act as a prophylactic agent for asthma  (46). Regarding the protective effects of safranal on asthma, an in vitro study of safranal (0.1, 0.5 and 1 mM) on cell viability and cytokine profile (IL-4, IL-10 and IFN-γ production) of phytohemagglutinin (PHA) stimulated and non-stimulated peripheral blood mononuclear cells (PBMC). Different safranal concentrations decreased the viability of stimulated PBMCs and inhibited the secretion of IL-10 and IFN-γ. Higher safranal concentrations significantly decreased the viability of non-stimulated cells. The effect of safranal on IL-4 secretion was less than that of dexamethasone. Safranal induced IFN-γ secretion by non-stimulated cells. In both non-stimulated and stimulated cells, the two higher concentrations of safranal had a significantly more marked effect on the IFN-γ/IL-4 ratio compared to the control and PHA-stimulated. suggesting its effects on the Th1/Th2 balance. Therefore, safranal showed therapeutic effects on some inflammatory disorders associated with Th1/Th2 imbalance such as asthma (47). Taken together, safranal besides its antitussive and muscle relaxant effects was found to act as a prophylactic agent in some respiratory tract diseases such as asthma (by increasing Th1/Th2 balance) and ALI (by anti-inflammatory and anti-oxidant effects). 

***Metabolic effects***

It has been reported that safranal could potentially reduce fasting blood glucose and hemoglobin A1c (HbA1c) levels and improve blood insulin levels significantly, while its effects on blood serum glutamic-oxaloacetic transaminase (SGOT), serum glutamic pyruvic transaminase (SGPT) and Cr levels were not significant (19). Samini and Bafandeh suggested that the anti-diabetic effects of safranal may be related to its anti-oxidant and anti-inflammatory activities (48). Also *in vivo* studies on rats indicated that safranal (2.5, 5, and 10 mg/kg) in combination with 5 mg/kg olanzapine effectively ameliorated olanzapine metabolic complications including elevated body weight, fasting blood glucose, triglyceride, and leptin levels, food intake, and decreased serum high-density lipoprotein (HDL) level (49). 

Another study in diabetic rats showed that safranal (0.25, 0.50, 0.75 mg/kg/day) treatment significantly lowered serum levels of oxidant agents (50). This investigation showed that MDA, NO, blood glucose, cholesterol, and triglycerides were significantly decreased by safranal. Moreover, safranal dose-dependently improved GSH levels and CAT and SOD activity. 

Moreover, safranal was shown to have beneficial effects on diabetes complications. Safranal (1 mg/kg in combination with insulin) not only modulated blood glucose in diabetic rats but also effectively improved diabetic neuropathy possibly via its anti-hyperglycemic, anti-oxidant, and antiapoptotic properties (51). Similarly, Hazman & Ovali revealed that since safranal can alleviate oxidative stress and inflammation, it could reduce diabetic complications in high-fat diet and/or STZ-induced diabetes in rats. In detail, 4 weeks of treatment with safranal significantly reduced TNF-α and IL-1β levels in type 2 diabetic rats (52). Also, treatments with safranal (0.025, 0.1, and 0.4 mg/kg) in combination with metformin (50 and 200 mg/kg) could improve learning and memory impairments in STZ-induced diabetic rats (53). 

The metabolic effects of safranal are not limited to diabetes mellitus and its complications as it was reported that the combination of N-acetylcysteine (NAC; 50 mg/kg, oral) and safranal (50 mg/kg, intraperitoneal) have beneficial effects on hyperthyroidism and its related brain damage in rats (54). These effects of safranal seem to be related to its anti-oxidative and anti-apoptotic properties.

***Effects on the gastrointestinal system***

Safranal exerts promising effects on different parts of the gastrointestinal (GI) system. 

Kareem and Zalzala (2021) reported that safranal (50 and 100 mg/kg/day) administered to rats with cyclophosphamide-induced liver injury ameliorated the liver function, declined MDA level, and increased GSH content and Nrf2 (nuclear factor erythroid 2-related factor 2) levels in a dose-dependent manner (55). Moreover, another study done in rats with liver ischemia-reperfusion- induced injury showed that safranal (100 mg/kg) administered 60 min after ischemia not only decreased plasma alanine aminotransferase (ALT) and aspartate aminotransferase (AST) but also improved histopathological characteristics and apoptotic activity (56). Also, safranal could potentially prevent age-induced liver injury in male rats; in this study, intraperitoneal injections of safranal (0.5 mg/kg day, for 1 month) produced a hormetic effect by induction of mild oxidative damage that leads to anti-oxidative enzyme activation (20). 

Using a rat model of indomethacin-induced gastric ulcer, the effects of lansoprazole (a proton pump inhibitor) and safranal (0.063, 0.25, and 1 mg/kg) were compared. Both agents controlled gastric volume and pH, decreased gastric ulcer area, produced gastric protection and improved histological changes and tissue biochemical changes; nevertheless, some beneficial effects of safranal were more impressive than lansoprazole (57).

Of note, the anti-inflammatory activity of safranal makes it a valuable agent in the treatment of inflammatory conditions such as inflammatory bowel syndrome (IBS). Low-dose (200 mg/kg, p.o.) and high-dose safranal (500 mg/kg, p.o) in DSS-induced colitis mice could suppress inflammatory processes and cryptos damage which leads to IBS clinical symptoms, via suppression of MAPK and NF-κB proteins in colonic tissues. Safranal could also reduce macrophage infiltration and IL-6 and TNF-α levels in colonic tissues  (26).

***Dermatologic effects***

Safranal could be used as a sun-protective agent which is more efficient than homosalate even at lower concentrations (58). In line with previous reports, an *in* *vitro* study found that solid lipid nanoparticle (SLN) formulations of safranal could be a beneficial carrier for topical delivery. In his study, SLN comprising 4% safranal demonstrated higher sun protective factor (SPF) values compared to other SLN formulations and 8% homosalate was used as the reference. In contrast with previous findings, the efficacy of safranal 4% or SLN-safranal 2% was not significantly higher than 8% homosalate. Beyond the sun-protective advantages of the SLN-safranal 4%, these formulations showed better hydration properties of the skin because of free-SLN formulations’ properties (59). Similarly, in another study by Sanju et al, it was found that safranal-loaded SLNs have approximately 88-99% efficacy as a sunscreen (60).

An *in vitro* study by Kumud and Sanju introduced safranal as a valuable agent to prevent photoaging with an SPF of 6.6 in 0.01% concentration. Their results confirmed that safranal is a better antisolar agent in comparison with homosalate at lower concentrations. Furthermore, the half-maximal inhibitory concentration (IC_50_) value of anti-elastase, anti-collagenase, and anti-hyaluronidase activity of safranal was 43.6, 9.4, and 70 µg/ml, respectively. Overall, safranal’s dermal enzyme inhibitory activities, substantial SPF, and remarkable anti-oxidant potential make it a novel antiaging bioactive compound (61).

***Effects on the central nervous system***

Saffron exhibits anti-oxidant activity in different neurological disorders including neurodegenerative and psychological disorders (62-64). Some of these pharmacological benefits were related to safranal content. 

*Anti-anxiety effects*

Saffron is known for its anti-anxiety properties. Anti-anxiety effects of the acute and chronic crocin-safranal mixture (CSM) in mice were evaluated using an elevated plus-maze test. The acute injection of CSM increased the time percentage spent in the open arm compared to the control group. Also, CSM at 0.68 mg/kg in the acute administration significantly increased the time percentage spent in the open arm compared to the same chronic dose. Therefore, CSM at all injected doses demonstrated anti-anxiety effects and the highest effect was observed at 4.08 mg/kg in the acute administration (65).

*Anticonvulsant effects*

Nanostructured lipid vehicles (NLV) carrying safranal have shown a significant effect, which was greater than sodium valproate, on treating generalized epilepsy both in the pilocarpine (PILO) and maximal electroshock (MES) induced acute seizure in mice. This anti-convulsant effect was dose-dependent, however, the maximal effect was observed at 100 mg/kg for PILO-induced seizures and 300 mg/kg for MES acute seizures (66). These findings were in line with our previous knowledge, but some detailed differences in terms of safranal dosage were determined which may be due to the different methods for inducing seizures. In another study, lamotrigine high-molecular weight micelles and safranal liposomal preparations significantly delayed the onset of clonic, myoclonic and tonic convulsions induced by strychnine (67).

*Neuroprotective effects*

Considering its anti-inflammatory and anti-apoptotic effects, safranal is expected to exert significant neuroprotection. In this regard, in traumatic spinal cord injured rats, safranal showed anti-inflammatory, anti-apoptotic and edema-attenuating effects, particularly at 100 mg/kg. Safranal inhibited spinal cord injury-induced upregulation of p38 MAPK (an anti-inflammatory effect) and the pro-apoptotic factor Bax, decreased cytokines IL-1β and TNF-αand suppressed the expression of aquaporin-4(AQP-4) protein due to its edema-attenuating effect (68). 

Also, in a rat model of Huntington’s disease induced by 3- nitropropionic acid (3-NP), safranal at doses of 0.75, 1.5, and 3 mg/kg remarkably prevented the increase of nitrite and MDA levels and reduced GSH content, and CAT and SOD activities. Furthermore, safranal-modified 3-NP induced changes in body weight, rotarod activity, number of vacuous chewing movements, and locomotor activity (69).

Moreover, in a model of oxidative damage induced by quinolinic acid (QA) in rat hippocampus, safranal at a low dose (72.75 mg/kg) could not reduce QA-induced damage, but at high concentrations, it could prevent lipid peroxidation, restore thiol redox, reduce DNA damage, and with its anti-oxidant and neuroprotective properties could prevent neurodegenerative disorders (70).

Neuroprotective effects of safranal were examined in OLN-93 cells that were pre-treated with safranal (0.1, 10, 50, 100, 200, 500 mM) for 2 hours and then with glutamic acid (16mM) or quinolinic acid (8mM) as a toxic agent for 24 hours; results showed that safranal has a neuroprotective effect by inhibition of oxidative stress parameters (i.e. reduction of ROS and MDA level) (71).

Safranal anti-oxidant activities have a positive effect on brain health and due to these properties, they can prevent oxidative damage caused by aging. An *in vivo* study showed that safranal could increase GSH, SOD and GST levels in the brain of old rats and decrease lipid peroxidation, thus exerting a beneficial effect on age-related brain damage (72) 

*Effects on memory*

In a rat model of Alzheimer’s disease (AD) induced by intra-hippocampal micro-injection of amyloid-beta (Aβ1–40), safranal (0.025, 0.1, and 0.2 ml/kg/day, orally for a week) effects on learning and memory were assessed; findings showed that safranal modified the hippocampal levels of MDA, ROS, protein carbonyl, IL-6, IL-1β, NF-kB, TNF-α, apoptotic biomarkers, glial fibrillary acidic protein (GFAP), DNA fragmentation, myeloperoxidase (MPO) as well as acetylcholinesterase (AChE) activity, and prevented CA1 neuronal loss. It ameliorated mitochondrial membrane potential (MMP) and SOD activity without remarkable effect on GSH content, CAT activity, or nitrite level. Furthermore, the rats’ cognition in novel-object discrimination, Y-maze, passive avoidance, and 8-arm radial arm maze tasks dose-dependently were improved (73). 

Another study evaluated safranal (2.5 and 5 μM) and donepezil (10 and 20 μM) effects on oxidative damage and toxicity caused by Aβ and hydrogen peroxide (H_2_O_2_) in PC12 cells as a proper Alzheimer’s cell damage model. According to the results, Aβ induced the apoptotic pathway through the PI3K/AKT pathway and the MAPK/ERK, and safranal had protective effects against Aβ-induced apoptosis in PC12 cells (74). Another study indicated that safranal protects against AD by decreasing lysozyme fibril accumulation in amyloid-related pathologies (75).

*Effects on Parkinson’s disease*

The potential effects of safranal on rotenone-induced Parkinson’s disease (PD) have been evaluated in an *in vitro* study in which, safranal decreased cellular apoptosis, ROS generation and expression of kelch-like-ECH-associated protein-1 (keap1) by promoting nuclear translocation of Nrf2. Nrf2 anti-oxidant downstream enzymes genes including glutathione-s -transferase, NADPH-quinone oxidoreductase 1 (NQO1), glutamate-cysteine ligase catalytic subunit (GCLC) and heme-oxygenase 1 (HO-1) were also induced by safranal. This study suggests that safranal has a protective effect on neurotoxicity induced by rotenone via induction of the Nrf2 signaling pathway. (76)

Safranal effects on dopaminergic neuron growth were investigated *in vitro* and *in vivo*; *in vitro*, one-week treatment with 20 and 100 ng/ml safranal could significantly increase tyrosine hydroxylase and dopamine transporter. Moreover, *in vivo* results determined that mentioned doses of safranal for 4 weeks not only increased the survival rate of the model rats but also significantly induced dopamine secretion and transplanted neural stem cell growth (77).

Safranal inhibited alpha-synuclein (α-syn) fibrillation/aggregation and induced dis-aggregation of pre-formed α-syn fibrils. These mechanisms are driven by hydrophobic interactions between safranal and the protein and may justify the therapeutic effect of safranal on various neurodegenerative diseases such as Parkinson’s disease (78).

*Effects on ischemia*

It was reported that safranal can significantly increase the total sulfhydryl content and anti-oxidant capacity in a model of focal cerebral ischemia after medial cerebral artery occlusion (MCAO). Thus, it decreases infarct volume, hippocampal cell loss, behavioral neuron deficiency due to ischemia, nerve cell loss and oxidative damage in the brain. Importantly, safranal provided more effective protection against ischemia at higher doses (145 mg/kg) compared to lower doses (72.5 mg/kg) (79). Furthermore, *in vitro*, safranal plus curcumin and thymoquinone could inhibit cell death induced by glucose/serum deprivation in PC12, indicating the potential of this natural combination against cerebral ischemia and neurodegenerative diseases (80). Another study revealed that pretreatment with safranal (40–160 μM) markedly reduced oxygen-glucose deprivation-induced cell death, oxidative stress and apoptosis in PC12 (81).

*Effects on tremor*

The effect of safranal on tremors induced by intraperitoneal injection of harmaline (30 mg/kg) has been investigated. The test group received safranal (0.1, 0.3, and 0.5 ml/kg) intraperitoneally 10 min before harmaline administration (preventive study) or 10 min after the onset of tremor (treatment study). Latency of onset, duration and tremor intensity were studied. Safranal at doses 0.1 and 0.3 ml/kg but not at 0.5 ml/kg decreased tremor duration and intensity. Safranal did not affect the latency of tremors. So, low-dose safranal has suppressive and protective effects on harmaline-induced tremors (Table 1) (82).

***Cytotoxic effects***

Safranal plays a critical role as a cytotoxic agent but the underlying mechanism is still unclear. *In vitro*, cytotoxic effects of safranal were mediated through binding to a tubulin dimer and changing its structure by hydrophobic reactions (83). Safranal also plays a role in the suppression of neuroblastoma cells (N2A) by inhibiting the overgrowth of neuroblastoma cells via proapoptotic and cytotoxic activity (84-86). Safranal also inhibited DNA and RNA synthesis and colony formation. Besides, it induced cell death via apoptosis in PC-3 cells in a dose- and time-dependent manner. These results suggest that the cytotoxic effect of safranal may help in preventing human prostate cancer (87).

In addition, safranal inhibited the proliferation of colo-205 cancer cells in a dose-dependent manner; this cytotoxic effect was indued through the reduction of MMP and ROS, leading to apoptosis of the cells. Also, it stopped the G2/M cell cycle, suppressed PI3K/AKT/mTOR signaling pathway and induced Bax expression in parallel with the reduction of Bcl-2 expression (88).

Safranal was reported to repress cell invasion and migration in oral squamous cell carcinoma by modulating the expression of epithelial-mesenchymal transition (EMT) proteins such as E-cadherin and vimentin in HSC-3 cells which are essential for the invasion and migration of cancer cells (89). 

*In vitro* and *silico*, safranal could inhibit *Bcr-Abl* gene expression in K562 cells similar to imatinib mesylate used in the treatment of chronic myeloid leukemia (CML). These data suggest that safranal may play a therapeutic role with high potential in the treatment of CML (90).

In another *in vitro* study, safranal exerted mild but effective anti-proliferative properties by attaching the cellular microtubules and secondary changes in its structure. Safranal reduced the viability of HeLa cells in a concentration-dependent manner. This study shows that safranal, not mainly through changing the structure of microtubules, but via disruption of the secondary structure of tubulin and interference with the microtubule reassembly potential has a targeted anti-proliferative effect (91).

Therefore, safranal possesses anti-proliferative effects that are induced through proapoptotic and cytotoxic activities on tubulin dynamism, and inhibition of RNA and DNA synthesis and Bcr-Abl gene expression in different cancerous cell lines. 

***Anti-nociceptive and anti-inflammatory effects***

In an *in vivo* study, the anti-inflammatory and antinociceptive effects of safranal and diclofenac were investigated at various doses. After injection of carrageenan, these agents prevented edema, cold allodynia, mechanical allodynia, hyperalgesia and neutrophilic infiltration in the paw tissues (92). According to this study, first, local biphasic edema (early and late phase) in the paw tissues was induced by injection of carrageenan (100 µl, 2%). It has been reported that safranal has dose-dependent effects in suppressing paw edema with no effect at 0.25 mg/kg. Safranal (0.5 mg/kg) suppressed only the late phase of edema and increasing the dose suppressed both late and early edema (92) (93). Cyclooxygenase inhibition has been suggested to play a considerable role in the anti-inflammatory and antinociceptive function of safranal (94-96, 92).

In another *in vivo* study, Tamdan Fard et al found that safranal 0.25 mg/kg could only significantly reduce the second phase of face rubbing induced by upper lip injection of formalin in rats. Nevertheless, safranal 0.5 mg/kg reduced both phases of pain. Also, sub-analgesic doses of safranal could produce significant analgesic effects when co-administered with sub-analgesic doses of morphine and diclofenac, thus, safranal was able to enhance the antinociceptive effects of diclofenac and morphine (97).

Anti-allodynia effect of the safranal was found to be mainly due to its suppression of glial activation and proinflammatory cytokines production in the central nervous system. In detail, safranal (0.1 mg/kg, i.p.) attenuated the pain sensitivity, inhibited inflammatory cytokines (TNF-α and IL-1β), and decreased glial activation markers (GFAP and OX-42) as the markers of the mechanical allodynia development in spinal nerve transection in rats (98). Moreover, another experiment showed that safranal and vitamin E similarly suppressed cold and mechanical allodynia caused by sciatic nerve injury and improved Wallerian degeneration and nerve atrophy while reducing MDA levels (99).

***Effects on eyes***

In a study, the IRS-PI3K-PDK1/2-AKT-BAD signaling pathway was reported as one of the mechanisms of choroidal neovascularization (CNV ) and the role of safranal in inhibiting this signaling pathway and CNV was evaluated *in vivo* and *in vitro*. *In vitro*, safranal exhibited a concentration-dependent inhibitory effect on oxidative stress in human choroidal microvascular endothelial cells (HCVECs) compared to the control group. *In vivo*, safranal on day 21 after laser-induced CNV formation, caused significantly less fluorescein leakage compared to the control rats. Also, the thickness of the CNV sections in the safranal group was less than that of the control group (100). 

The neuroprotective effects of safranal on retinal degeneration were shown to be mediated by inhibiting photoreceptor cells (101, 102), A recent study investigated the protective effect of safranal on cataracts in rats and found that subcutaneous injection of sodium selenite in rats produced considerable cataracts and increased the level of lipid peroxidation (MDA) but decreased the level of GSH and Nrf2 protein. In contrast, safranal had the opposite effect and it showed favorable anti-cataract effects by increasing Nrf2 protein expression and GSH level. Also, by reducing MDA and soluble protein levels in the lens and due to its anti-oxidant effect, safranal presented potent anticataractogenic effects. However, clinical trials are needed to evaluate safranal effects on cataractogenesis in humans (103).

***Other effects***

Antigenotoxic effects of safranal have been evaluated in mice. Mice were given safranal (0.025-0.25 ml/kg, orally), then exposed to genotoxic agents including gamma radiation (2Gy), urethan (800 mg/kg) and procarbazine (60 mg/kg). Measurement of genotoxic damage was done using the bone marrow micronucleus test. A significant reduction of micro-nucleated polychromatic erythrocytes in the bone marrow of safranal pre-treated mice was observed (104).

In another study, the effect of safranal on erythrocyte osmotic fragility and some hematological parameters was evaluated in CCL_4_-intoxicated rats. Results showed that safranal ameliorated erythrocyte osmotic fragility induced by CCL_4_ in rats after 7 days, while it did not affect the hematological parameters (105).

Moreover, studies on the anti-microbial effects of safranal especially methicillin-resistant *Staphylococcus aureus* (MRSA) showed that safranal diepoxide activity against MRSA is greater than safranal and monoepoxide (106). It has been suggested that the inhibition of *Escherichia coli *cell growth by safranal may be associated with its inhibitory effect on ATP synthase (107).

***Patents related to pharmacological effects of safranal***

Different formulations containing safranal have been patented worldwide for the prevention and/or treatment of different diseases such as liver cancer, sleep disorder, depression, cognitive disorder, obesity, premenstrual syndrome (PMS), etc (Table 2).

***Toxicity***

Acute and sub-acute safranal toxicity was investigated in mice and rats and 21 days after administration. Hematological, biochemical and pathological changes were assessed for sub-acute toxicity. Results showed lethal dose 50% (LD_50_) values for intraperitoneal safranal of 1.5 ml/kg for male rats, 1.48 ml/kg for male mice and 1.88 ml/kg for female mice; however oral LD_50_ values were 5.53 ml/kg for male rats, 21.42 ml/kg for male mice and 11.42 ml/kg for female mice. Sub-acute toxicity of 21-day administration of oral safranal (0.1, 0.25 or 0.5 mL/kg/day) to male rats showed that this exposure led to reduced levels of triglyceride, alkaline phosphatase, cholesterol, hematocrits, platelets, red blood cell (RBC) counts and hemoglobin. Furthermore, results showed that safranal increased serum LDH and BUN levels. Histological evaluation revealed no toxicity in the heart, liver or spleen but pathological changes were observed in the kidneys and lungs. According to the calculated LD_50_ values, acute intraperitoneal administration of safranal is of low toxicity and the acute oral administration route was found non-toxic in both rats and mice. However, sub-acute exposure induced biochemical and hematological changes (29).

Co-exposure of safranal and saffron aqueous extract in acute and sub-acute exposures was assessed in rats. Safranal (1 and 2 ml/kg, IP) with a saffron aqueous extract (25-100 mg/kg, IP) was given to rats to examine acute toxicity, then 1 and 4 days after the exposure, mortality percentage was assessed. Rats were divided into the following 6 groups for sub-acute toxicity: group 1 safranal ( 0.2 ml/kg, IP), groups 2, 3 and 4 safranal with saffron aqueous extracts (5,10 and 20 mg/kg, IP) and groups 5 and 6 paraffin with normal saline (as safranal and saffron aqueous extract solvents, respectively). In sub-acute exposure (21 days), biochemical markers were assessed. Results showed that 4 days of co-treating rats with safranal and saffron aqueous extract decreased mortality significantly. Sub-acute toxicity data showed that saffron (10 mg/kg) increased the survival of rats as no mortality was seen during the sub-acute study. IP administration of safranal in sub-acute toxicity (0.2 ml/kg/day) raised triglyceride, BUN and ALT significantly, however, co-treatment of safranal and saffron aqueous extracts (5 and 10 mg/kg) improved all biochemical markers and safranal toxicities (108). So, it can be suggested that the administration of the whole herb (i.e. saffron) causes fewer adverse effects than the isolated ingredient, safranal.

The toxic effects of safranal on mice fetuses were investigated. Pregnant BALB/c mice were divided into the following 3 experimental and control groups; 2 groups were given IP injections of safranal (0.75 and 0.225 ml/kg) on gestational days 5 to 16 (GDs). The Control group received paraffine as the solvent of safranal. On the 18th GD, dams were separated and collected for further analysis. The macroscopic assessments of embryos were done for external malformations and common fetal and maternal markers were checked. Double skeletal staining with alcian blue and alizarin red for selected fetuses was performed. Embryos of experimental groups showed significantly decreased weight and length compared to the control group. Moreover, growth retardation, mandible and calvaria with minor skeletal malformations were observed. The most frequently seen malformation among embryos was a minor skeletal one. This investigation showed that safranal could induce fetal malformations when administered to pregnant mice; so more research for understanding the mechanisms of malformations induced following maternal exposure to safranal should be considered (109).

In another study, safranal immunotoxicity was evaluated. Safranal has injected IP (0.1, 0.5 and 1 ml/kg) for 3 weeks in BALB/c mice and then histopathological changes in the spleen and bone marrow, as well as cellularity of the spleen, cytokine production, hemagglutination (HA) titer, lymphocyte proliferation and delayed type of hypersensitivity (DTH) response, were assessed. Spleen cellularity was not significantly different compared to the control. Moreover, safranal at the given doses did not significantly affect hematological parameters such as HA titer, DTH, lymphoproliferation response or cytokine production of free spleen cells. Despite some toxicology studies which showed that safranal is more toxic compared to other constituents of saffron, it was observed that safranal was not toxic to the immune system as no humoral and cellular immune responses were observed following exposure to the compound (110).

Furthermore, an *in vitro* study on HCC cells (hepatocellular carcinoma) showed that protein destabilization resulting in hypoxanthine accumulation which in turn induces apoptosis via ROS production may be considered the primary mechanism of safranal cytotoxicity, because of hypoxanthine (111).

**Table 1:** Protective effects of safranal on the central nervous system

**Effect**	**Study design**	**Safranal dose/duration of study**	**Results**	**Reference**
**Anti-anxiety**	In Vivo, mice	2.04 and 4.08 mg/kg	↑ Time spent in the open arm of EPM by acute doses of CSM compared to control ↑ Time spent in the open arm of EPM by 0.68 mg/kg of CSM compared to the chronic dose	(65)
**Anti-convulsant**	In Vivo (induced by PTZ, PILO and MES in mice)	NLV safranal (100 mg/kg in PILO and PTZ induced) (300 mg/kg in MES-induced)	↑ Latency to generalized seizure, ↓ Highest seizure stages ↓ Number of generalized seizures The effect was comparable to sodium valproate ↓ EEG spectra power in PILO-induced seizure ↑ Electroconvulsive threshold in MES-induced seizure Delayed the kindling rate of progress and the time it took to reach generalized seizures in PTZ-induced seizure	(66)
**Anti-convulsant**	**In Vivo (strychnine-induced convulsion)**	IV. administration of lamotrigine HMW micelles and safranal niosomal preparations	Delayed the onset of clonic, myoclonic and tonic convulsions	(67)
**Neuroprotective Effect**	**In Vivo (SCI in rat)**	100 mg/kg	↓ BAX ↓ IL-1β ↓ TNF-α ↓ p38 MAPK	(68)
**Neuroprotective effect**	In Vivo (3-NP induced Huntington’s disease in rat)	0.75, 1.5, and 3 mg/kg for two weeks	↓ Nitrite, MDA, ↑ SOD, catalase, GSH ↑ Body weight ↓ Number of VCM	(69)
**Neuroprotective Effect**	**In Vivo (QA induced oxidative damage in rat hippocampus**	291 mg/kg, IP	↓ LPO and ↓ Oxidative DNA damage ↑ Hippocampal thiol redox ↑ Antioxidant status	(70)
**Neuroprotective Effect**	In Vitro (GA and QA induced oxidative stress in OLN-93 cells)	(0.1, 10, 50, 100, 200, 500 mM) for 2 hours	↓ ROS ↓ MDA	(71)
**Neuroprotective Effect**	In Vivo ( 2, 10 and 20 months old rats)	0.5 mg/kg/ day for 1 month	↑ GSH, SOD, GST ↓ LPO	(72)
**Anti-Alzheimer's disease**	In Vivo (Aβ-induced Alzheimer's in rats)	0.025, 0.1, and 0.2 ml/kg for 7 days	↓ MDA, ROS, IL-6, protein carbonyl, IL-1β, NF-kB, TNFα, caspase 3, DNA fragmentation, GFAP, MPO, AChE ↑ SOD, MMP	(73)
**Anti-Alzheimer's disease**	In Vitro ( Aβ and H2O2 induced oxidative stress )	2.5 and 5 μM for 120 min	↓ ROS ↓ PI3K/AKT, MAPK/ERK	(74)
**Anti-Alzheimer's disease**	In Vitro (Hen egg white lysozyme	Various concentrations	Inhibition in the rate of amyloid formation	(75)
**Anti-Parkinson's disease**	In Vitro (Rotenone-induced Parkinson's disease)	Various concentrations	↓ Apoptosis ↓ ROS ↓ keap1 ↑ Nrf2	(76)
**Anti-Parkinson’s disease**	In vivo (safranal treated rat neural stem cells were administrated into PD rat models induced by 6-OHDA)	20 and 100 ng/ml safranal for one and 4 week	↑ Tyrosine hydroxylase after one week ↑ Dopamine transporter after one week ↑ Dopamine secretion after one and 4 weeks ↑ Transplanted neural stem cells growth after 4 weeks	(77)
**Anti-Parkinson's disease**	In Vitro	Various concentrations	Inhibition α-syn fibrillation/aggregation Dis-aggregation pre-formed α-syn fibrils	(78)
**Anti-ischemia**	In vivo, rat(MCAO)	145 mg/kg for 0, 3, and 6 h after reperfusion	↓ Infarct volume ↓Hippocampal cell loss ↓Behavioral neuron deficiency ↓Oxidative damage in the brain	(79)
**Anti-ischemia**	In vitro ( glucose/serum deprivation in PC12)	50 µg/ml	Safranal+curcumin+thymoquinone inhibit cell death	(80)
**Anti-tremor**	In Vivo (harmaline-induced tremor in mice)	0.1 and 0.3 ml/kg	↓ Tremor duration ↓ Tremor intensity	(82)

EPM: elevated plus maze; CSM: crocin-safranal mixture; PILO: pilocarpine; EEG: electroencephalogram; MES: maximal electroshock; PTZ: pentylenetetrazol; NLV: nanostructured lipid vehicles; HMW: high molecular weight; SCI: Spinal cord injury; IL1β: interleukin 1β; TNF-α: tumor necrosis alpha; MDA: malondialdehyde; SOD: superoxide dismutase; GSH: glutathione; VCM: vacuous chewing movement; LPO: lipid peroxidation; ROS: reactive oxygen specious; GA: glutamic acid; QA: quinolinic acid; PD: Parkinson disease; α-syn: alpha-synoclein; MPO: myeloperoxidase; MMP: matrix metalloproteinase; AChE: acetylcholinesterase

**Table 2:** Patents containing safranal pharmacological effects

**Patent number**	**Patent title**	**Compositions**	**Pharmacological usage**	**Reference**
WO2015124318	The edible composition including safranal, crocin, picrocrocin and a vitamin B complex for treating the initial phase of depression	Safranal, crocin, picrocrocin and vitamin B (including vitamin B1, B2, B3, B6, B8, B9 and B12)	Treating the initial phase of depression	(112)
US20100028464	Use of saffron and/or safranal and/or crocin and/or picrocrocin and/or derivatives thereof as a satiety agent for the treatment of obesity	Saffron and/or safranal and/or crocin and/or picrocrocin and/or derivatives	Treatment of obesity	(113)
AU2019264659A1	Combination therapy for cancer	Safranal and optionally topoisomerase-1 inhibitors	Treating liver cancer	(114)
US20200253890A1	**Suppression and Inhibition of CDC25B with Safranal-Based Formulations**	A therapeutically effective amount of a composition having safranal	Treating, suppressing, or reducing the severity of hyperproliferative diseases	(115)
JP2020132625A	**Combination therapy of safranal and sorafenib for liver cancer**	Safranal or a pharmaceutically acceptable prodrug thereof and sorafenib	Treatment of liver cancer	(114)
US20200276133A1	**Prevention of liver cancer with safranal-based formulations**	A prophylactically effective amount of safranal	Prevention of liver cancer	(116)
EP3446678A1	Saffron extract and its use for the prevention of mood disorders related to depression	Safranal: 0.03% -1 % dry weight; Crocins: 3.48 % dry weight [ trans-crocin-4 (major isomer), trans-crocin-3, trans-crocin-2', cis-crocin-4, trans crocin-2, and trans-crocin-1 isomers ]	prevention of mood disorders related to depression	(117)
US9707203B1	**Methods of killing bacteria and preventing or treating bacterial infection with oxidation products of safranal and methods of synthesizing safranal epoxides**	An effective amount of at least one oxidation product of safranal	Killing bacteria and preventing or treating bacterial infection	(118)
US20220218779	Composition comprising extracts of *Eucommia ulmoides*, *Crocus sativus* and/or *Magnolia officinalis* and the use thereof in the treatment of sleep disorders.	A mixture consisting of an extract of *Eucommia ulmoides *and saffron comprising safranal and an extract of *Magnolia officinalis *and/or *Magnolia champaca *comprising honokiol	Treatment of sleep disorders.	(119)
WO2020229412A1	Treatment of premenstrual syndrome and/or menstrual discomfort with a composition comprising vicenin-2	vicenin-2 in association with one or more further ingredients such as rosmarinic acid, clerodadienols, crocin, safranal, apigenins, vitamin D and magnesium	Treatment of premenstrual syndrome (PMS) and/or menstrual discomfort	(120)
IN201911007661	Broad spectrum sun protective topical formulation	Safranal entrapped spherical solid lipid nanoparticles, colored zinc oxide, colored pearl powder, *Pterocarpus santalinus* extract, gum acacia, *Aloe vera* gel, shea butter, egg oil, other emollients, antioxidant, emulsifier, chelating agent, preservative, skin nourishing and skin protective agents	Sun protective	(121)
WO2018108879A1	**Composition for use in treating dysphoria, depression and/or mood swings relating to premenstrual syndrome (pms)**	A pollen and pistil extract derived from the *Graminae and/or Pinacea* family, preferably from plant material originating from *Zea mays* L.*, Secale cereale *L.*, Dactylis glomerata *L.*, Pinus sylvestris *L*.* and at least one second active ingredient being a source of safranal and/or (picro)crocin.	**Treating dysphoria, depression and/or mood swings relating to PMS**	(122)
WO2022128915A1	**Extract of at least one plant of the species crocus sativus comprising a high crocin content and a low safranal content, and cosmetic use thereof as an antioxidant**	At least one plant of the species *Crocus sativus* extracts comprising 20% by dry weight of one or more crocins and less than 0.08% by dry weight of safranal	Antioxidant	(123)
WO2021209455A1	**Composition for improving cognitive function**	A mixture of catechin and/or epicatechin, safranal and crocetin in aglycone and/or glycosylated form	**Improving cognitive function**	(124)

**Figure 1:**
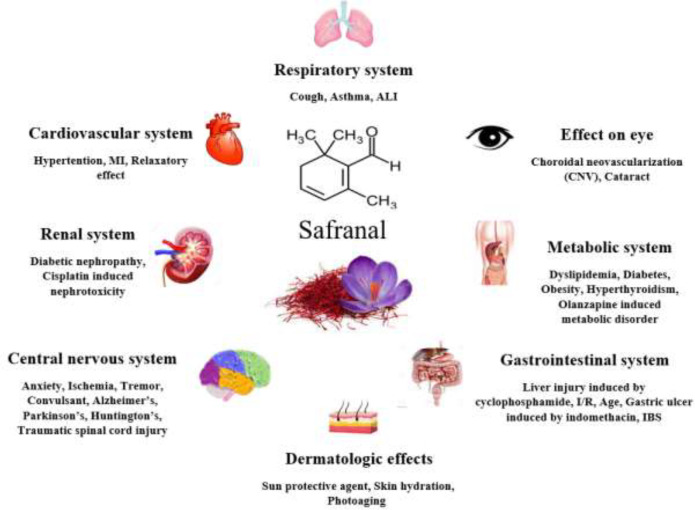
Some important therapeutic effects of safranal

ALI: acute lung injury; MI: myocardial infarction; I/R: ischemia-reperfusion; IBS: Irritable bowel syndrome

## Conclusion

Safranal, the main component of volatile oils of saffron, possesses different pharmacological effects including anti-oxidant, anti-inflammatory, antihypertensive, antiischemic, anti-asthmatic, antianxiety, anticonvulsant, analgesic, antinociceptive, and cytotoxic activities. Safranal has been found to show cardioprotective, nephroprotective, neuroprotective, gastrointestinal protective and lung protective effects (Figure 1). The present article reviewed studies published between 2010 and 2022 on the pharmacological effects of safranal. According to the literature, most of the studies are related to the effects of safranal on the CNS and present antianxiety, analgesic, anticonvulsant, antiischemic, anti-tremor, and memory-enhancing functions as well as protective effects on neurodegenerative disorders such as Alzheimer’s, Parkinson’s and Huntington’s diseases. Moreover, the protective effects of safranal on metabolic syndrome, diabetic nephropathy and ALI have been shown. Different mechanisms including anti-oxidant, anti-inflammatory, muscle relaxation, antiapoptotic, and regulatory effects on the expression of genes and proteins involved in different signaling pathways related to oxidative stress, inflammation, apoptosis, proliferation, etc., are among safranal pharmacological effects. Besides, some patents for the prevention and/or treatment of different diseases such as liver cancer, sleep disorder, depression, cognitive disorder, obesity, PMS, etc were found. Toxicological studies revealed that acute intraperitoneal administration of safranal is of low toxicity and acute oral administration is considered non-toxic in both rats and mice. Sub-acute exposure to safranal resulted in biochemical and hematological changes. Moreover, immunological studies showed no toxicity (i.e. no humoral or cellular immune responses) for safranal on mice’s immune system. Based on the documents, safranal is considered a promising therapeutic agent although more clinical studies are needed to verify its beneficial effects in humans.

## Authors’ Contributions

H H study conception, design and supervision of the research; BBM R critical revision of the paper, supervision of the research; D E and A A M G and M S preparation of original draft. All authors have agreed to the contents and approved the final version for publication.

## Conflicts of Interest

The authors declare that they have no conflicts of interest.
